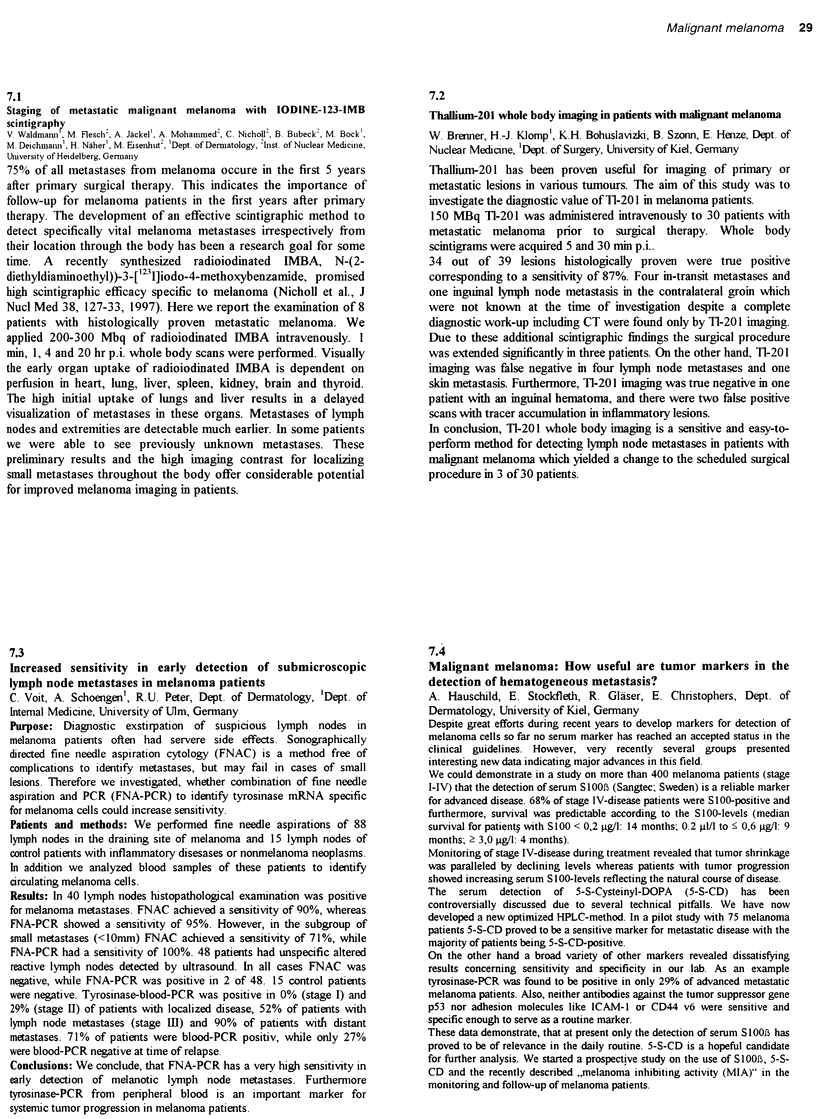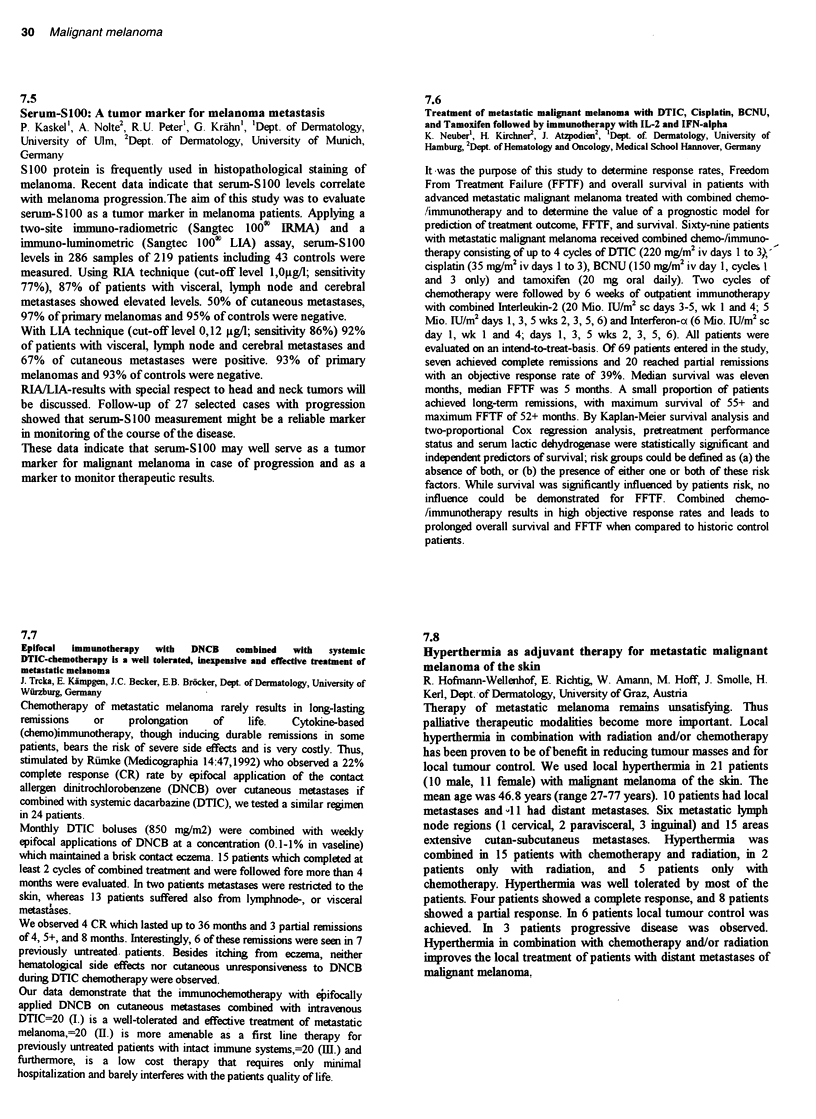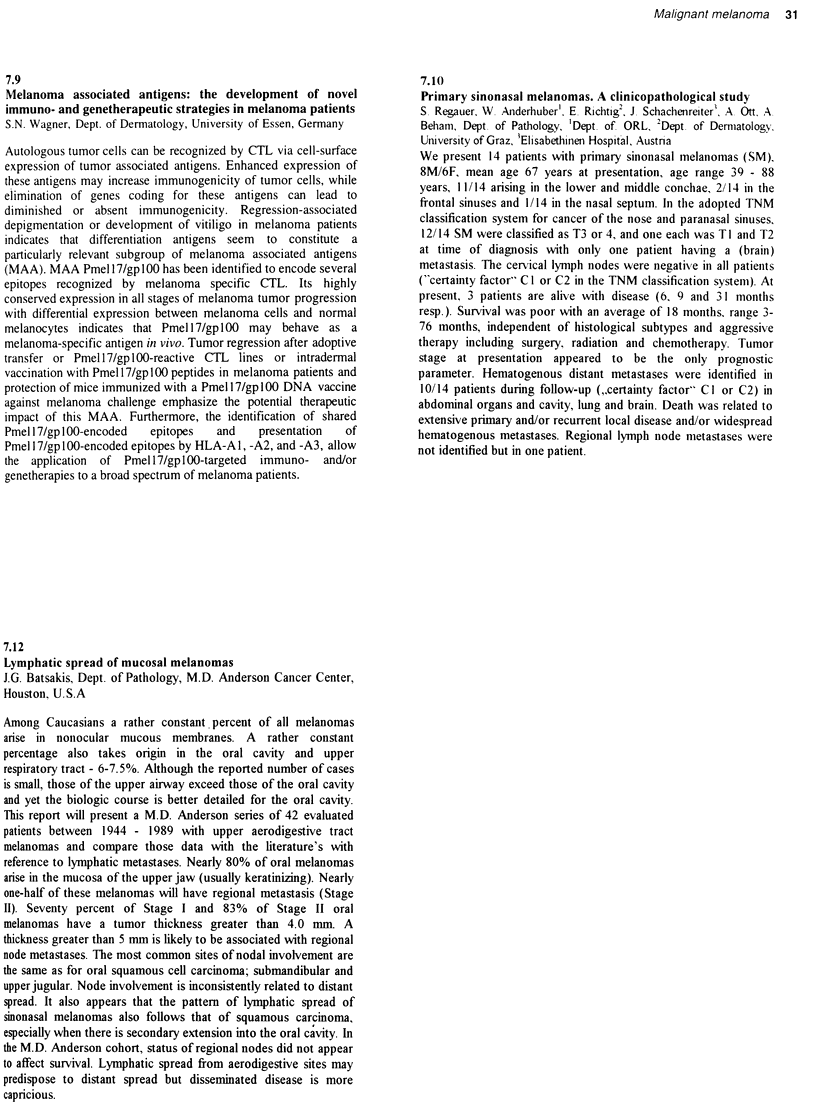# Malignant melanoma

**Published:** 1998

**Authors:** 


### Staging of metastatic malignant melanoma with IODINE-123-IMB scintigraphy

V. Waldmann[1], M. Flesch[2], A. Jäckel[1], A. Mohammed[2], C. Nicholl[2], B. Bubeck[2], M. Bock[1], M. Deichmann[1], H. Näher[1], M. Eisenhut[2], [1]Dept. of Dermatology, [2]Inst. of Nuclear Medicine, University of Heidelberg, Germany

75% of all metastases from melanoma occure in the first 5 years after primary surgical therapy. This indicates the importance of follow-up for melanoma patients in the first years after primary therapy. The development of an effective scintigraphic method to detect specifically vital melanoma metastases irrespectively from their location through the body has been a research goal for some time. A recently synthesized radioiodinated IMBA, N-(2-diethyldiaminoethyl))-3-[$^{123}$I]iodo-4-methoxybenzamide, promised high scintigraphic efficacy specific to melanoma (Nicholl et al., J Nucl Med 38, 127-33, 1997). Here we report the examination of 8 patients with histologically proven metastatic melanoma. We applied 200-300 Mbq of radioiodinated IMBA intravenously. 1 min, 1, 4 and 20 hr p.i. whole body scans were performed. Visually the early organ uptake of radioiodinated IMBA is dependent on perfusion in heart, lung, liver, spleen, kidney, brain and thyroid. The high initial uptake of lungs and liver results in a delayed visualization of metastases in these organs. Metastases of lymph nodes and extremities are detectable much earlier. In some patients we were able to see previously unknown metastases. These preliminary results and the high imaging contrast for localizing small metastases throughout the body offer considerable potential for improved melanoma imaging in patients.

### 7.2

### Thallium-201 whole body imaging in patients with malignant melanoma

W. Brenner, H.-J. Klomp[1], K.H. Bohuslavizki, B. Szonn, E. Henze, Dept. of Nuclear Medicine, [1]Dept. of Surgery, University of Kiel, Germany

Thallium-201 has been proven useful for imaging of primary or metastatic lesions in various tumours. The aim of this study was to investigate the diagnostic value of Tl-201 in melanoma patients.

150 MBq Tl-201 was administered intravenously to 30 patients with metastatic melanoma prior to surgical therapy. Whole body scintigrams were acquired 5 and 30 min p.i..

34 out of 39 lesions histologically proven were true positive corresponding to a sensitivity of 87%. Four in-transit metastases and one inguinal lymph node metastasis in the contralateral groin which were not known at the time of investigation despite a complete diagnostic work-up including CT were found only by Tl-201 imaging. Due to these additional scintigraphic findings the surgical procedure was extended significantly in three patients. On the other hand, Tl-201 imaging was false negative in four lymph node metastases and one skin metastasis. Furthermore, Tl-201 imaging was true negative in one patient with an inguinal hematoma, and there were two false positive scans with tracer accumulation in inflammatory lesions.

In conclusion, Tl-201 whole body imaging is a sensitive and easy-to-perform method for detecting lymph node metastases in patients with malignant melanoma which yielded a change to the scheduled surgical procedure in 3 of 30 patients.

### 7.3

### Increased sensitivity in early detection of submicroscopic lymph node metastases in melanoma patients

C. Voit, A. Schoengen[1], R.U. Peter, Dept. of Dermatology, [1]Dept. of Internal Medicine, University of Ulm, Germany

**Purpose:** Diagnostic exstirpation of suspicious lymph nodes in melanoma patients often had servere side effects. Sonographically directed fine needle aspiration cytology (FNAC) is a method free of complications to identify metastases, but may fail in cases of small lesions. Therefore we investigated, whether combination of fine needle aspiration and PCR (FNA-PCR) to identify tyrosinase mRNA specific for melanoma cells could increase sensitivity.

**Patients and methods:** We performed fine needle aspirations of 88 lymph nodes in the draining site of melanoma and 15 lymph nodes of control patients with inflammatory disesases or nonmelanoma neoplasms. In addition we analyzed blood samples of these patients to identify circulating melanoma cells.

**Results:** In 40 lymph nodes histopathological examination was positive for melanoma metastases. FNAC achieved a sensitivity of 90%, whereas FNA-PCR showed a sensitivity of 95%. However, in the subgroup of small metastases (<10mm) FNAC achieved a sensitivity of 71%, while FNA-PCR had a sensitivity of 100%. 48 patients had unspecific altered reactive lymph nodes detected by ultrasound. In all cases FNAC was negative, while FNA-PCR was positive in 2 of 48. 15 control patients were negative. Tyrosinase-blood-PCR was positive in 0% (stage I) and 29% (stage II) of patients with localized disease, 52% of patients with lymph node metastases (stage III) and 90% of patients with distant metastases. 71% of patients were blood-PCR positiv, while only 27% were blood-PCR negative at time of relapse.

**Conclusions:** We conclude, that FNA-PCR has a very high sensitivity in early detection of melanotic lymph node metastases. Furthermore tyrosinase-PCR from peripheral blood is an important marker for systemic tumor progression in melanoma patients.

### 7.4

### Malignant melanoma: How useful are tumor markers in the detection of hematogeneous metastasis?

A. Hauschild, E. Stockfleth, R. Gläser, E. Christophers, Dept. of Dermatology, University of Kiel, Germany

Despite great efforts during recent years to develop markers for detection of melanoma cells so far no serum marker has reached an accepted status in the clinical guidelines. However, very recently several groups presented interesting new data indicating major advances in this field.

We could demonstrate in a study on more than 400 melanoma patients (stage I-IV) that the detection of serum S100ß (Sangtec; Sweden) is a reliable marker for advanced disease. 68% of stage IV-disease patients were S100-positive and furthermore, survival was predictable according to the S100-levels (median survival for patients with S100 < 0,2 µg/l: 14 months; 0.2 µl/l to ≤ 0,6 µg/l: 9 months; ≥ 3,0 µg/l: 4 months).

Monitoring of stage IV-disease during treatment revealed that tumor shrinkage was paralleled by declining levels whereas patients with tumor progression showed increasing serum S100-levels reflecting the natural course of disease.

The serum detection of 5-S-Cysteinyl-DOPA (5-S-CD) has been controversially discussed due to several technical pitfalls. We have now developed a new optimized HPLC-method. In a pilot study with 75 melanoma patients 5-S-CD proved to be a sensitive marker for metastatic disease with the majority of patients being 5-S-CD-positive.

On the other hand a broad variety of other markers revealed dissatisfying results concerning sensitivity and specificity in our lab. As an example tyrosinase-PCR was found to be positive in only 29% of advanced metastatic melanoma patients. Also, neither antibodies against the tumor suppressor gene p53 nor adhesion molecules like ICAM-1 or CD44 v6 were sensitive and specific enough to serve as a routine marker.

These data demonstrate, that at present only the detection of serum S100ß has proved to be of relevance in the daily routine. 5-S-CD is a hopeful candidate for further analysis. We started a prospective study on the use of S100ß, 5-S-CD and the recently described „melanoma inhibiting activity (MIA)" in the monitoring and follow-up of melanoma patients.

### 7.5

### Serum-S100: A tumor marker for melanoma metastasis

P. Kaskel[1], A. Nolte[2], R.U. Peter[1], G. Krähn[1], [1]Dept. of Dermatology, University of Ulm, [2]Dept. of Dermatology, University of Munich, Germany

S100 protein is frequently used in histopathological staining of melanoma. Recent data indicate that serum-S100 levels correlate with melanoma progression.The aim of this study was to evaluate serum-S100 as a tumor marker in melanoma patients. Applying a two-site immuno-radiometric (Sangtec 100® IRMA) and a immuno-luminometric (Sangtec 100® LIA) assay, serum-S100 levels in 286 samples of 219 patients including 43 controls were measured. Using RIA technique (cut-off level 1,0µg/l; sensitivity 77%), 87% of patients with visceral, lymph node and cerebral metastases showed elevated levels. 50% of cutaneous metastases, 97% of primary melanomas and 95% of controls were negative.

With LIA technique (cut-off level 0,12 µg/l; sensitivity 86%) 92% of patients with visceral, lymph node and cerebral metastases and 67% of cutaneous metastases were positive. 93% of primary melanomas and 93% of controls were negative.

RIA/LIA-results with special respect to head and neck tumors will be discussed. Follow-up of 27 selected cases with progression showed that serum-S100 measurement might be a reliable marker in monitoring of the course of the disease.

These data indicate that serum-S100 may well serve as a tumor marker for malignant melanoma in case of progression and as a marker to monitor therapeutic results.

### 7.6

### Treatment of metastatic malignant melanoma with DTIC, Cisplatin, BCNU, and Tamoxifen followed by immunotherapy with IL-2 and IFN-alpha

K. Neuber[1], H. Kirchner[2], J. Atzpodien[2], [1]Dept. of Dermatology, University of Hamburg, [2]Dept. of Hematology and Oncology, Medical School Hannover, Germany

It was the purpose of this study to determine response rates, Freedom From Treatment Failure (FFTF) and overall survival in patients with advanced metastatic malignant melanoma treated with combined chemo-/immunotherapy and to determine the value of a prognostic model for prediction of treatment outcome, FFTF, and survival. Sixty-nine patients with metastatic malignant melanoma received combined chemo-/immunotherapy consisting of up to 4 cycles of DTIC (220 mg/m$^2$ iv days 1 to 3), cisplatin (35 mg/m$^2$ iv days 1 to 3), BCNU (150 mg/m$^2$ iv day 1, cycles 1 and 3 only) and tamoxifen (20 mg oral daily). Two cycles of chemotherapy were followed by 6 weeks of outpatient immunotherapy with combined Interleukin-2 (20 Mio. IU/m$^2$ sc days 3-5, wk 1 and 4; 5 Mio. IU/m$^2$ days 1, 3, 5 wks 2, 3, 5, 6) and Interferon-α (6 Mio. IU/m$^2$ sc day 1, wk 1 and 4; days 1, 3, 5 wks 2, 3, 5, 6). All patients were evaluated on an intend-to-treat-basis. Of 69 patients entered in the study, seven achieved complete remissions and 20 reached partial remissions with an objective response rate of 39%. Median survival was eleven months, median FFTF was 5 months. A small proportion of patients achieved long-term remissions, with maximum survival of 55+ and maximum FFTF of 52+ months. By Kaplan-Meier survival analysis and two-proportional Cox regression analysis, pretreatment performance status and serum lactic dehydrogenase were statistically significant and independent predictors of survival; risk groups could be defined as (a) the absence of both, or (b) the presence of either one or both of these risk factors. While survival was significantly influenced by patients risk, no influence could be demonstrated for FFTF. Combined chemo-/immunotherapy results in high objective response rates and leads to prolonged overall survival and FFTF when compared to historic control patients.

### 7.7

### Epifocal immunotherapy with DNCB combined with systemic DTIC-chemotherapy is a well tolerated, inexpensive and effective treatment of metastatic melanoma

J. Trcka, E. Kämpgen, J.C. Becker, E.B. Bröcker, Dept. of Dermatology, University of Würzburg, Germany

Chemotherapy of metastatic melanoma rarely results in long-lasting remissions or prolongation of life. Cytokine-based (chemo)immunotherapy, though inducing durable remissions in some patients, bears the risk of severe side effects and is very costly. Thus, stimulated by Rümke (Medicographia 14:47,1992) who observed a 22% complete response (CR) rate by epifocal application of the contact allergen dinitrochlorobenzene (DNCB) over cutaneous metastases if combined with systemic dacarbazine (DTIC), we tested a similar regimen in 24 patients.

Monthly DTIC boluses (850 mg/m2) were combined with weekly epifocal applications of DNCB at a concentration (0.1-1% in vaseline) which maintained a brisk contact eczema. 15 patients which completed at least 2 cycles of combined treatment and were followed fore more than 4 months were evaluated. In two patients metastases were restricted to the skin, whereas 13 patients suffered also from lymphnode-, or visceral metastases.

We observed 4 CR which lasted up to 36 months and 3 partial remissions of 4, 5+, and 8 months. Interestingly, 6 of these remissions were seen in 7 previously untreated patients. Besides itching from eczema, neither hematological side effects nor cutaneous unresponsiveness to DNCB during DTIC chemotherapy were observed.

Our data demonstrate that the immunochemotherapy with epifocally applied DNCB on cutaneous metastases combined with intravenous DTIC=20 (I.) is a well-tolerated and effective treatment of metastatic melanoma,=20 (II.) is more amenable as a first line therapy for previously untreated patients with intact immune systems,=20 (III.) and furthermore, is a low cost therapy that requires only minimal hospitalization and barely interferes with the patients quality of life.

### 7.8

### Hyperthermia as adjuvant therapy for metastatic malignant melanoma of the skin

R. Hofmann-Wellenhof, E. Richtig, W. Amann, M. Hoff, J. Smolle, H. Kerl, Dept. of Dermatology, University of Graz, Austria

Therapy of metastatic melanoma remains unsatisfying. Thus palliative therapeutic modalities become more important. Local hyperthermia in combination with radiation and/or chemotherapy has been proven to be of benefit in reducing tumour masses and for local tumour control. We used local hyperthermia in 21 patients (10 male, 11 female) with malignant melanoma of the skin. The mean age was 46.8 years (range 27-77 years). 10 patients had local metastases and 11 had distant metastases. Six metastatic lymph node regions (1 cervical, 2 paravisceral, 3 inguinal) and 15 areas extensive cutan-subcutaneus metastases. Hyperthermia was combined in 15 patients with chemotherapy and radiation, in 2 patients only with radiation, and 5 patients only with chemotherapy. Hyperthermia was well tolerated by most of the patients. Four patients showed a complete response, and 8 patients showed a partial response. In 6 patients local tumour control was achieved. In 3 patients progressive disease was observed. Hyperthermia in combination with chemotherapy and/or radiation improves the local treatment of patients with distant metastases of malignant melanoma.

### 7.9

**Melanoma associated antigens: the development of novel immuno- and genetherapeutic strategies in melanoma patients**

S.N. Wagner, Dept. of Dermatology, University of Essen, Germany

Autologous tumor cells can be recognized by CTL via cell-surface expression of tumor associated antigens. Enhanced expression of these antigens may increase immunogenicity of tumor cells, while elimination of genes coding for these antigens can lead to diminished or absent immunogenicity. Regression-associated depigmentation or development of vitiligo in melanoma patients indicates that differentiation antigens seem to constitute a particularly relevant subgroup of melanoma associated antigens (MAA). MAA Pmel17/gp100 has been identified to encode several epitopes recognized by melanoma specific CTL. Its highly conserved expression in all stages of melanoma tumor progression with differential expression between melanoma cells and normal melanocytes indicates that Pmel17/gp100 may behave as a melanoma-specific antigen *in vivo*. Tumor regression after adoptive transfer or Pmel17/gp100-reactive CTL lines or intradermal vaccination with Pmel17/gp100 peptides in melanoma patients and protection of mice immunized with a Pmel17/gp100 DNA vaccine against melanoma challenge emphasize the potential therapeutic impact of this MAA. Furthermore, the identification of shared Pmel17/gp100-encoded epitopes and presentation of Pmel17/gp100-encoded epitopes by HLA-A1, -A2, and -A3, allow the application of Pmel17/gp100-targeted immuno- and/or genetherapies to a broad spectrum of melanoma patients.

### 7.10

**Primary sinonasal melanomas. A clinicopathological study**

S. Regauer, W. Anderhuber[1], E. Richtig[2], J. Schachenreiter[3], A. Ott, A. Beham, Dept. of Pathology, [1]Dept. of ORL, [2]Dept. of Dermatology, University of Graz, [3]Elisabethinen Hospital, Austria

We present 14 patients with primary sinonasal melanomas (SM), 8M/6F, mean age 67 years at presentation, age range 39 - 88 years, 11/14 arising in the lower and middle conchae, 2/14 in the frontal sinuses and 1/14 in the nasal septum. In the adopted TNM classification system for cancer of the nose and paranasal sinuses, 12/14 SM were classified as T3 or 4, and one each was T1 and T2 at time of diagnosis with only one patient having a (brain) metastasis. The cervical lymph nodes were negative in all patients ("certainty factor" C1 or C2 in the TNM classification system). At present, 3 patients are alive with disease (6, 9 and 31 months resp.). Survival was poor with an average of 18 months, range 3-76 months, independent of histological subtypes and aggressive therapy including surgery, radiation and chemotherapy. Tumor stage at presentation appeared to be the only prognostic parameter. Hematogenous distant metastases were identified in 10/14 patients during follow-up („certainty factor" C1 or C2) in abdominal organs and cavity, lung and brain. Death was related to extensive primary and/or recurrent local disease and/or widespread hematogenous metastases. Regional lymph node metastases were not identified but in one patient.

### 7.12

**Lymphatic spread of mucosal melanomas**

J.G. Batsakis, Dept. of Pathology, M.D. Anderson Cancer Center, Houston, U.S.A

Among Caucasians a rather constant percent of all melanomas arise in nonocular mucous membranes. A rather constant percentage also takes origin in the oral cavity and upper respiratory tract - 6-7.5%. Although the reported number of cases is small, those of the upper airway exceed those of the oral cavity and yet the biologic course is better detailed for the oral cavity. This report will present a M.D. Anderson series of 42 evaluated patients between 1944 - 1989 with upper aerodigestive tract melanomas and compare those data with the literature's with reference to lymphatic metastases. Nearly 80% of oral melanomas arise in the mucosa of the upper jaw (usually keratinizing). Nearly one-half of these melanomas will have regional metastasis (Stage II). Seventy percent of Stage I and 83% of Stage II oral melanomas have a tumor thickness greater than 4.0 mm. A thickness greater than 5 mm is likely to be associated with regional node metastases. The most common sites of nodal involvement are the same as for oral squamous cell carcinoma; submandibular and upper jugular. Node involvement is inconsistently related to distant spread. It also appears that the pattern of lymphatic spread of sinonasal melanomas also follows that of squamous carcinoma, especially when there is secondary extension into the oral cavity. In the M.D. Anderson cohort, status of regional nodes did not appear to affect survival. Lymphatic spread from aerodigestive sites may predispose to distant spread but disseminated disease is more capricious.